# Vaccination in the Light of the Royal British Commission

**Published:** 1898-02

**Authors:** M. R. Leverson

**Affiliations:** Fort Hamilton, L. I., N. Y.


### VACCINATION IN THE LIGHT OF THE ROYAL BRITISH COMMISSION.

M. R. Leverson, M. D., Fort Hamilton, L. I., N. Y.

(Continued from January No., p. 19.)

Q. 4,516. Dr. Cory defines "spontaneous cow-pox" as "the first case occurring in a herd that you cannot say has been communicated by man." Q. 4,517. He believes it has been so communicated; "but that is only an opinion."*

Q. 4,519 (Sir James Paget). "You say communicated by man. What is the man suffering from?" "I am referring to small-pox, though cow-pox can be communicated from human subjects having vaccine."

Q. 4,520. "In the case you had your own lymph derived from, you think it probable that some one with small-pox had milked the cow?" "There was small-pox very much about Laforet at the time." (See *post.* 4,578-9.)

Q. 4,521 (Dr. Collins). "Is that stated in the *Gazette Hebdomadaire de Medecine et Chirugie?*" "Yes; I believe so."

The modification "I believe so" was not made by Dr. Cory when giving his testimony on November 27th, 1889, but, as he admits at the next sitting, on December 4th, 1889 (Qs. 4,575-6), he added that qualification subsequently, and (Q. 4,578) it turns out that the *Gazette* says nothing of the kind!

Q. 4,577 (Dr. Collins). "On what grounds did you found

---

*In all the literature of vaccination and of cow-pox which I have been able to search, not one single case has been mentioned of small-pox being thus accidentally given to the cow. There is even very great doubt whether it has been so given by inoculation. But the cunning trick of Edward Jenner in giving to the cow-pox the name of "small-pox of the cow," though without the smallest fraction of evidence to support it, has created so strong a bias in the minds of most medical students and their teachers, followers of each other and of routine as they nearly all are, that in the teeth of evidence, men claiming to be men of science *assume* that some person suffering from small-pox gives that disease to the cow, producing in it the cow-pox!

the belief that the original source of that so-called spontaneous cow-pox was due to the inoculation from a milker suffering from small-pox?" "I only believed so at the time. It is my general belief that vaccinia is modified small-pox."

Q. 4,579. "So that beyond a general belief that cow-pox is referable in all cases to the inoculation of human small-pox, you are not aware of evidence which exists to connect the two in that particular outbreak?" "No; I am not."

And this is scientific testimony!

Q. 4,586 (By Dr. Collins). Dr. Cory was not aware that the Laforet (Dubreuilh) "lymph" had been discontinued in France, nor (Q. 4,587) that it had been criticised as "spurious," nor (Q. 4,588) did he know anything of Dr. Leyet's criticisms upon it, and added, "If it had been a spurious case we should have found it out ourselves." But as appeared from his answer to Q. 4,589, only "by the appearance of the vesicle." So that from Dr. Cory's statement it appears that the L. G. B. go ahead and insert into healthy blood the putrefying matter of a sore of some kind, they don't know what, and if disaster should result they would then know that it was "spurious."

A curious thing, indeed, comes out from Dr. Cory's testimony concerning this curious superstition. We have the L. G. B. using indiscriminately (1) the by-French-experts-rejected-Laforet-Dubreuilh spontaneous lymph as a source of the British supply, and (2) Dr. Simpson's small-pox-inoculated-producing cow-pox.*

According to the theories of the vaccinists these two sources of "pure calf-lymph" are diametrically opposed to one another. According to some of the theories both are spurious; that is what Jenner and Simon said; according to every theory yet put forward by the vaccinists, one or other of them is. Further (Q. 4,600), Dr. Cory, the propagafor of the "by-French-experts-rejected-Laforet-Dubreuilh

* This, according to the French authorities, is an *impossibility*.

lymph" among the unfortunate English and Scotch was actually ignorant of the notorious fact that one of the first cases inoculated with it in Paris terminated fatally!

Qs. 4,613-14. Dr. Cory admits that horses suffer from horse-pox as much as mares, while among bovines it is only found normally among cows, and explains it on the hypothesis that horses get the horse-pox when being shod through the farrier having small-pox.

Q. 4,616. He fancies he remembers something to the effect that at Bordeaux it was found that better success was obtained by the use of horse-pox than by that of cow-pox. The reader is referred to Dr. Crookshank's *History and Pathology of Vaccination* for an account of this filthy disease, which frequently assumes a character resembling glanders.

Q. 4,618. Dr. Cory attributes the rarity of cow-pox in England to the diminution of small-pox; but (Qs. 4,619-20) according to the report on the eruptive diseases of the teats and udders of cows of the Agricultural Department of the Privy Council for 1888 there seems to have been a large number of cases of cow-pox in various parts of the country, says Dr. Cory, "of what they said was cow-pox, I believe."

Q. 4,621. "Does any evidence exist to show that these cases were not cow-pox?" "Not that I know of."

Q. 4,623. His present contention is contrary to the conclusions arrived at by the Lyons Commission on the experiments conducted by M. Chaveau.

Qs. 4,624-5-6. Mr. Fleming, of the War Office, is an authority upon diseases of cattle and horses, and he states: "I will now venture to dispute every one of the arguments brought forward to prove that human variola and cow-pox are due to the same virus, or are the same disease."

Q. 4,627. The Irish Local Government Board in the year 1879 warned the Galway Board of Guardians against the use of lymph obtained by inoculating a cow with small-pox.

Q. 4,628-9. Dr. Warlomont is an authority upon animal vaccination. He contends that it is impossible to convert small-pox into cow-pox by the inoculation of the cow with small-pox.

Dr. Kleiss, in his paper published in the London *Lancet*, December 17th, 1887, says, page 1240, b, "I am afraid that as long as the present uncertainty as to the exact nature of the virus of vaccinia and variola remains, no clear experimental proof will be obtainable unless, etc. * * * as regards the first—that is, the nature of the virus, various assertions have been made from time to time without bringing us much nearer to the exact understanding."

F. Cohn, he said, many years ago ascertained the presence of micrococcus of the character of streptococcus in the lymph of variola and vaccinia. To the same effect Sanderson, Weigerband, Pohl, Pineas. Dr. Paul Guttman isolated by cultivation after the modern method three different varieties of micrococci from the lymph of vaccinia and variola, so also Dr. Buest, and "I show you here a streptococcus which is constantly present in human vaccinia," and in the same number of the London *Lancet*, page 1210, a, Dr. Klein says:

"It must be evident from these observations that the danger of scarlatinal infection from the disease in the cow is real, and that towards the study and careful supervision of this cow disease all efforts ought to be directed to check the spread of scarlet fever in man."

Q. 4,522. "Were you at all acquainted with the case of cow-pox in Wiltshire in 1887?" "No; I know there was a case spoken of by that name, but I do not know much about it."

Here begins an interesting inquiry regarding a case of "spontaneous cow-pox" discovered by Dr. Crookshank, at Cricklade, in Wiltshire, but the curious and contradictory testimony given by Dr. Cory will not be understood without some previous account of its discovery and of the strange controversy to which it gave rise.*

The Wiltshire case was seen and described by Dr. Crookshank in a report to the agricultural department of the Privy Council. The substance of this report was published in the London *Lancet* of December 17th, 1887. Dr. Buchanan and Dr. Cameron, as well as Dr. Crookshank, agreed that this Wiltshire case was true cow-pox. This Dr. Klein denied (London *Lancet*, December 17th and 24th, 1887, pp. 1232, 1240-2†), and later Dr. Buchanan denied that it was cow-pox, and called it dermatitis pustulosa, a disease unknown to the compilers of encyclopædias, to lexicographers (even medical), and to dermatologists, and seems to have been invented for the occasion by Dr. Buchanan. Now, bearing in mind Dr. Cory's distinct reference to the Gloucestershire lymph (Q. 4,316)), showing he had not forgotten that lymph but had it distinctly in his mind when testifying, let us continue the abstract of his testimony.

Qs. 4,522-6. On November 27th Dr. Cory states that the L. G. B. made inquiry into the Wiltshire lymph. Dr. Cory took a general interest in it. He did not receive any lymph from that case of cow-pox, but some calves were vaccinated by Dr. Klein at the calf lymph station after passing through the Wiltshire disease; he forgets whether any lymph from that case was brought to the national vaccine establishment.

On December 4th he states that he has ascertained that the L. G. B. did investigate it, and that Dr. Klein made experiments at Lamb's Conduit Street (the calf-lymph-station of

* The pathology of this Wiltshire cow-pox is given in Dr. Crookshank's *History and Pathology of Vaccination*, Vol. I, pp. 360–4; also in the London *Lancet*, December 17th, 1887, pp. 1208–1212.

† On referring to the *Lancet*, the reader will find frequent references to the Heudon Case and will be hard put to it to be sure whether Dr. Klein is speaking of the Wiltshire Case or of that at Heudon. This need not trouble him, for Dr. Klein says that the Heudon Case was the same as the Wiltshire one.

which Dr. Cory is director), and he believes there are notes of those experiments (Qs. 4,630-1).

Q. 4,632. "I took some of Dr. Klein's lymph down to St. Thomas's Hospital, and some children there were vaccinated with it."

Q. 4,633. "With a perfectly normal result?" "A drawing was taken of the arm, and it went through a perfectly normal course. In fact, the lymph used at St. Thomas's for three or four months was lymph derived from that source."

Q. 4,634 (Sir James Paget). "Was that lymph which Dr. Klein took from some of the Wiltshire cows?" "Yes; I understand from Dr. Klein that he obtained that lymph from cows in Wiltshire."

Q. 4,635. "What was the result of inoculating with it?" "It produced perfectly normal vaccine vesicles."

Q. 4,636 (Dr. Collins). "Did I correctly understand you to say that that lymph was, in the opinion of Dr. Klein, the true and not spurious cow-pox?" "I do not know what Dr. Klein's opinion was respecting the cows from which he took it."

Q. 4,638. "Will you give us your own view, from having watched the progress of vaccination, as to whether the results would be such as to enable you to class it as true and not spurious cow-pox?" "I should certainly class it as true and not as spurious cow-pox."

Q. 4,639. "Do you happen to know anything of the origin of that lymph?" "No, except that it was found in some eruptions on the udders of cows in Wiltshire, I believe."

Q. 4,640. "Did any evidence exist to connect the origin of that outbreak, either directly or indirectly, with small-pox?" "Not that I know of."

Q. 4,641. "Can you tell the commission whether a stock has been raised from that lymph, and is still being used?" "No; we have given it up at St. Thomas's Hospital now, but it was used for three or four months there."

Q. 4,643. "Do you think that the lymph derived from the French source was superior to that which was obtained in Wiltshire?" "No; the results.so far as I observed them were very much the same."

Q. 4,647 (Dr. Collins). "So that the only protection which cinated any of those children afterwards with other lymph?" "Not when they had the normal vesicles."

Q. 4,647 (Dr. Collins). "So that the only protection which those children would have would be that which would be given by the Wiltshire cow-pox?" "Yes; what I have spoken of as the cow-pox from Wiltshire."

At Q. 4,783 the subject is renewed, and Dr. Collins asked: "Are you aware that in a communication by Dr. Klein to the Local Government Board in 1887-8 he makes this statement: 'In the study of the Wiltshire disease proper I have already made some progress, but hardly sufficient to warrant an attempt to diagnose it as seen during life from other teat diseases'?" "I do not remember that." And (Q. 4,784) "He (*i. e.*, Dr. Klein) also states on page 214, 'In view of this second differentiation of a definite disease from among the mass of cow diseases that show sores on the teats the old division into true and spurious cow-pox has become manifestly insufficient. It is seen that the name "spurious cow-pox" has in all probability been used to cover a variety of sores having essential differences in nature, just as until the time of Jenner the name "cow-pock" had covered, along with various other things, the disease which we now know as vaccinia. But it is one thing to have learnt the essential nature of these sores in the cow that are concerned with vaccinia, or scarlatina, in the human subject, and another thing to affirm the distinguishing characters by which those sores may be recognized from other sores that once on a time laid claim to being equally with them cow-pox or spurious cow-pox. Our new discontent with the name spurious cow-pox does not at once give us a knowledge of the nature of those sores which remain on the list, and we are now learning that there are many different kinds of such sores.' So that apparently the pathology of cow-pox is rather obscure even at the present time?" "That is Dr. Klein's opinion."

Q. 4,785 (Sir James Paget). "Was it after that that he sent you the lymph from the Wiltshire disease?" "He brought up the lymph I have referred to the Lamb's Conduit Street station, and we tried the lymph on a calf."

Q. 4,786. "When did he do that; was it at the same time that he made that report or afterwards?" "He must have made that report before, I think."

Q. 4,787. "He made the report before you tried the lymph?" "Yes."

Q. 4,788 (Chairman). "It was in the year 1887, as I understand, that you tried this lymph?" "So far as I can remember it was in 1888."

Q. 4,789 (Sir James Paget). "And the effects produced by that virus led you to the complete belief that the Wiltshire disease is true cow-pox vaccinia?" "Yes; that is to say, I believe that the disease of the cow from whom that lymph was taken was true cow-pox."

After all this Dr. Cory makes a foot-note to Q. 4,630 and Q. 4,783, as follows:

In revising this proof I observe repeated reference (Qs. 4,630 to 4,647, and Qs. 4,783 to 4,789) to lymph from Wiltshire used by Dr. Klein at Lamb's Conduit. I have written to the Chairman of the Commission, stating, in reference to these questions and answers, that the lymph to which I was here referring came not from Wiltshire, but from Alderley, in Gloucestershire, R. C.

On May 7, 1890, Dr. Cory was recalled in consequence of the letter above referred to, and for the sake of convenience I will here present Dr. Cory's explanation:

Q. 8,862 (Dr. Collins). "Was the lymph which was employed derived from a cow at Alderley?" "Yes, I believe so."

Q. 8,863. "Prior to employing that lymph for the vaccination of children did you investigate the source from which it came?" "It was given to me by Dr. Klein, and I used it on calves before I vaccinated children with it."

Q. 8,864. "You were not aware at the time of using it whence it was derived?" "No; Dr. Klein gave it to me, and the confusion arose in this way: Dr. Klein spoke to me of it as Wiltshire lymph, whereas it came from Alderley on the borders of Gloucestershire."

Qs. 8,865-6. He is quite clear that no children were vaccinated directly or indirectly with the Wiltshire disease.

Q. 8,867. The Alderley lymph was derived from a vesicle on the hand of a milkmaid, aged twenty. He refers the Commission to the report of Dr. Klein, in the report of the medical officer of the L. G. B. for 1888, for details as to the source of lymph.

Q. 8,868. "Did the girl on whose hand the vesicle appeared present three good marks of infantile vaccination?" "Do you mean the girl from whom the lymph was derived?"

Q. 8,869. "Yes?" "I do not know anything about the source of the lymph, except that Dr. Klein gave it to me."

Q. 8,870. "Is it not stated in this report to which you have drawn my attention?" "Yes; it may be."

Q. 8,871. "So that that stock of lymph was derived from what was practically a re-vaccination with casual cow-pox?" "I must refer you to Dr. Klein for the source of the lymph. It produced typical vaccine vesicles upon the calves, and also upon the children vaccinated with it."

Q. 8,872. Dr. Klein stated upon page 384 of the report to which Dr. Cory had called the attention of the committee that the girl from whose hand the stock of lymph was raised had three good primary vaccination marks. So that this Alderley lymph was a case of retro-vaccination from a girl who had been vaccinated and whose "cow-pox," therefore, was a case of re-vaccination. With this the calf is inoculated and a stock of lymph raised from the sore thus produced. But it is to be remembered that the Local Government Board have heretofore been most emphatic in prohibiting the use of the virus of re-vaccination either to raise or continue a stock or for arm-to-arm vaccination.

Q. 8,873. "Do you recommend the use of lymph derived from re-vaccination for raising a stock?" "Recommend? No."

Q. 8,874. Dr. Cory, from the appearances observed by himself upon the calves and children, came to the conclusion that the case was one of true and not of spurious vaccine.

Q. 8,875. "You would attach great importance to the use of the true and not of the spurious vaccine?" "Yes; one protects from small-pox, and the other does not."

Qs. 8,876-7. Dr. Cory agrees with Jenner that "we must set off by impressing the idea that there will be no end to cavil and controversy until it be defined with precision what is and what is not cow-pox," and that "the true has many imitations by the spurious, all being called the cow-pox."

Q. 8,878. "You agree that there are varieties of the disease which have hitherto been confounded?" "Yes."

Q. 8,879 (Sir James Paget). "Or were confounded in Jenner's time; not necessarily confounded now?" "No; they are more separated now."

Q. 8,880 (Dr. Collins). "But are they all distinctly differentiated now?" "I cannot say that they are all distinctly differentiated now, but two or three have been separated distinctly one from the others."

Q. 8,881. "Can you refer me to any previous report to that to which you have drawn my attention for the first time to-day, in which a serious attempt has been made to discriminate the variety of diseases of the udder and the teats of cows which had been previously classed as cow-pox?"

"There was a recent report of the Veterinary Department, which published some observations, but I am not sure that it undertook such discrimination."

Q. 8,882 (Mr. Bradlaugh). "This report has only been circulated the last few days?" "That is all."

Q. 8,883 (Dr. Collins). "Could you describe to the Commission the appearance of what you would consider absolutely indicative of the true as opposed to the various forms of spurious cow-pox?" "That is rather a difficult question to answer offhand; but the ordinary course run by the disease, as we see it at Lamb's Conduit Street, would be this: that you make the insertion of the lymph one day, and then on about the fourth day you first observe some local irritation of the part which is more or less a line of inflammation; on the fifth day it becomes distinctly vesicular, and we can take the lymph from it; and on the sixth day it is further advanced; and about the fourteenth or fifteenth day the crust is fully formed, and generally soon separates; it is a broad, hard, dark brown, mahogany-colored crust."

Q. 8,884. "Would the vaccinia material which produced that series of phenomena which you have described be such as to enable you to define that as true cow-pox?" "It would leave so strong an impression upon my mind if I saw it (not if others described it) that I should not mind using the lymph for the vaccination of children; and if I observed the same course in the children as is usual with vaccinia, then I should be quite convinced that it was vaccinia. Before adopting it as stock one would, of course, like a further proof that the fresh lymph was a protection against small-pox; and that proof we have in the lymph we are using at Lamb's Conduit Street."

Q. 8,885. "How so?" "A good many of the nurses of the small-pox hospital were re-vaccinated with the lymph."

Q. 8,886. "What lymph?" "The Lamb's Conduit Street lymph."

Q. 8,887. "Not the Alderley?" "No, not the Alderley; and the lymph in current use protected* the nurses from small-pox."

Q. 8,888. Dr. Cory in his work on cow-pox and horse-pox quotes observations tending to show that on various occasions stocks of lymph have been raised from an eruptive disease on the cow, which, from the description, appears to be similar to cattle plague.

Q. 8,889. In the same work (p. 19), quoting from Mr. Macpherson, he says that from crusts taken from cattle affected with the disease known as the gotee or mhata in India, eleven native children were inoculated (one of them successfully), in whom appeared a vesicle having all the characters of true vaccine. From this vesicle two children were successfully vaccinated, the symptomatic fever being very severe. From these two five others were successfully vaccinated, and the stock thus established was regularly continued. Dr. Cory now remarks that Dr. Seaton, commenting on the above story, says: "From these facts it is not to be doubted that a case of cow-

---

*Marvelous is the logic of Dr. Cory in the assumptions here implied. Although their absurdity has been over and over again demonstrated in the writings of the anti-vaccinationists, the grotesqueness of the argument may, perhaps, be presented in a clearer light by putting it into syllogistic form:

Some nurses do not take small-pox;
Some of the nurses who do not take small-pox are vaccinated.
Therefore, all nurses who are vaccinated do not take small-pox.
Or, Some *A's* are *B's;*
Some *B's* are *C's;*
Therefore, all *C's* are *B's*.

Even if we were to assume (contrary to the fact) that *all nurses* who do not take small-pox are vaccinated, or *all B's* are *C's*, it yet would not follow that all *C's* are *B's* any more than because all cows are animals we might thence conclude that all animals are cows; yet this is the logic of the vaccinationists. Unfortunately, logic forms no part of the curriculum of medical students either in England or the United States.

Koch's celebrated argumentation, by which he sought to establish a basis for the bacterio-germ theory of disease, rests on a like logical blunder to that above displayed.

pox in the cow had been met with; but what is to be doubted is that the gotee, the malignant disease above referred to, was the source of infection."

Q. 8,890. "Do the appearances quoted by Mr. Macpherson agree with the appearances of vaccinia or cattle plague in the cow?" "Vaccinia, I should think, judging by results."

Q. 8,891. "It is thus described, according to Mr. Lamb, whom you quote, 'The animals which were at first affected had been for a day or two previously dull and stupid; they were afterwards seized with cough, and much phlegm collected in their mouths and fauces. The animals had at this time no inclination for food. There is a discharge of saliva from the mouth; then follow universal tremor, and great heat of the head, chest, and body, as far back as the loins, while the hindquarters are cold; the whole body then becomes hot, and the animals suffer from intense thirst. The mouth and fauces appear to be the principal seat of the disease, being in some instances one mass of ulceration. On the fifth day the eruption appears about the udder, sometimes only a few pustules, and at other times they were numerous and confluent, but the result of the attack does not appear to depend much on the eruption. Whether the pustules are numerous or rare, the disease is nearly always fatal, and unless measures are taken to separate the diseased from the healthy, it speedily runs throughout the whole herd, sparing few.' Is that a description of cow-pox?" "Certainly not; but there may have been two diseases confounded there. You have the vaccinia in one and the cattle plague in the other. We saw in England that the cattle plague was quite a separate disease from vaccinia."

Q. 8,892. "But I think you told us that you had never seen cow-pox upon a cow?" "No; not the so-called spontaneous disease."

Q. 8,893. "You are satisfied that from the results of inoculation upon calves that you are able to discriminate the true from the spurious cow-pox?" "Yes."

Q. 8,894. "And are you satisfied that those appearances arising in cattle, although stated to produce the vesicle from which stocks of lymph were raised, arise from a disease improperly described as cow-pox?" "No. I do not think the prevalent cattle disease referred to by Mr. Macpherson was improperly described, nor described as cow-pox, but that it was rather cattle plague. Dr. Seaton refers to this series of experiments, and he says there is no doubt that somehow or other veritable vaccinia had been obtained."

Q. 8,895. "Do you think it possible that local appearances of so-called vaccinia can be produced by the inoculation of any other material than that derived from the special form of cow-pox, such as that at Alderley, which you describe as the true cow-pox?" "I think there are many eruptions which may resemble it. The figures given in the description of the accidental inoculation of cattle plague on men are not dissimilar to the vaccine vesicle; but that would surely show its difference when inoculated fresh upon another animal, and continued through a series."

Q. 8,896. "Have you inoculated cattle plague upon calves?" "No."

Q. 8,897. "Do not the local results of inoculation with true cow-pox vary considerably according to the period of the vesicle at which the lymph is taken?" "Not in the earlier days of it; they vary after the sixth day a good deal; the lymph is then much weaker, producing earlier vesicles and earlier areola."

Q. 8,898. "Are not the appearances which are regarded as typical vaccinia to some extent the result of the cultivation of the vaccine?" "I think not at all largely. You may alter the lymph slightly by cultivation, but to a very slight extent."

Q. 8,899. "The stock you employ habitually at Lamb's Conduit Street was supplied by Dr. Dubreuilh?" "Yes."

Q. 8,900. "Are you aware that it was stated that the earlier results of inoculation with that lymph were not typical, and that it was only in the fourth remove that they became apparently true?" "That is so, so far as I can ascertain respecting its earlier transmissions, but I was not aware of it when you asked me the question on the occasion of my previous examination."

Q. 8,901. "Have you formed any opinion in your own mind as to the nature of the Wiltshire disease?" "No."

Q. 8,902. "Or of the Alderley?" "Yes; that it is vaccinia."

Q. 8,903. "You do not consider the fact that the milkmaid, aged twenty, developed a large vesicle half an inch wide in spite of having three good infantile vaccination marks an argument against its being true cow-pox?" "You can undoubtedly have re-vaccination within twenty years of primary vaccinia in some individuals almost like a primary vaccination."

Q. 8,904. "With a vesicle half an inch wide?" "Yes; the size of the vesicle depends upon the surface of the skin that is inoculated."

Q. 8,905. Refers to Dr. Klein whether it has been ascertained that the Alderley outbreak was derived from the inoculation of small-pox upon the cow through the milker. Dr. Cory did not see any of the first removes of the Alderley lymph.

Q. 8,906. Dr. Cory has given it as his opinion that true cow-pox never arises except by the direct contact with the human variola.

Q. 8,907. He has not endeavored to ascertain whether that was so in this particular instance.

Q. 8,908. (Chairman). "Would it be possible to ascertain it with any absolute certainty?" "No; it would not."

Q. 8,909. "A person might be in contact with some one having small-pox without having been conscious of it himself?" "Yes."

Q. 8,910 (Dr. Collins). "Is there any statement in the report that small-pox had been about in the village of Wootton-under-Edge where the diseased cow was discovered?" "I do not know."

