# Corrections in the February Number

**Published:** 1898-04

**Authors:** 


### CORRECTIONS IN THE FEBRUARY NUMBER.

The following corrections and additions should be made in the abstract of the testimony taken by the Royal (Br.) Commission on Vaccination in the February issue of THE HOMŒOPATHIC PHYSICIAN:

Page 56, second paragraph, first line, for "Dr. Kleiss" read *Dr. Klein.*

Page 59. Instead of the second paragraph read:

Q. 4,646 (Dr. Bristowe). "May I ask whether you re-vaccinated any of those children afterwards with other lymph?" "Not when they had the normal vesicles."

In the article by Dr. Klein, quoted on page 56 of THE HOMŒOPATHIC PHYSICIAN for February, 1898, Dr. Klein quotes F. Cohn, Pohl Pineas, Dr. Paul Guttmann, and Dr. Buist as authorities for the statement that the streptococcus is constantly present in vaccinia.

Dr. Walter Reed, U. S. A., in a paper read before the District of Columbia Medical Society, June 5th, 1895, says the same thing with regard to all the vaccine virus examined by him. All these are advocates of vaccination, and their reports need to be remembered when we hear people talk of "pure calf lymph."

